# Drug-Induced Trafficking of P-Glycoprotein in Human Brain Capillary Endothelial Cells as Demonstrated by Exposure to Mitomycin C

**DOI:** 10.1371/journal.pone.0088154

**Published:** 2014-02-04

**Authors:** Andreas Noack, Sandra Noack, Andrea Hoffmann, Katia Maalouf, Manuela Buettner, Pierre-Olivier Couraud, Ignacio A. Romero, Babette Weksler, Dana Alms, Kerstin Römermann, Hassan Y. Naim, Wolfgang Löscher

**Affiliations:** 1 Department of Pharmacology, Toxicology, and Pharmacy, University of Veterinary Medicine Hannover, Hannover, Germany; 2 Department of Trauma Surgery, Hannover Medical School, Hannover, Germany; 3 Department of Physiological Chemistry, University of Veterinary Medicine Hannover, Hannover, Germany; 4 Institute of Functional and Applied Anatomy, Hannover Medical School, Hannover, Germany; 5 INSERM, U1016, Institut Cochin, Paris, France; 6 Centre National de la Recherche Scientifique (CNRS), UMR8104, Paris, France; 7 Université René Descartes, Paris, France; 8 Department of Biological Sciences, The Open University, Milton Keynes, United Kingdom; 9 Weill Medical College of Cornell University, New York, New York, United States of America; 10 Center for Systems Neuroscience, Hannover, Germany; Hungarian Academy of Sciences, Hungary

## Abstract

P-glycoprotein (Pgp; ABCB1/MDR1) is a major efflux transporter at the blood-brain barrier (BBB), restricting the penetration of various compounds. In other tissues, trafficking of Pgp from subcellular stores to the cell surface has been demonstrated and may constitute a rapid way of the cell to respond to toxic compounds by functional membrane insertion of the transporter. It is not known whether drug-induced Pgp trafficking also occurs in brain capillary endothelial cells that form the BBB. In this study, trafficking of Pgp was investigated in human brain capillary endothelial cells (hCMEC/D3) that were stably transfected with a doxycycline-inducible MDR1-EGFP fusion plasmid. In the presence of doxycycline, these cells exhibited a 15-fold increase in Pgp-EGFP fusion protein expression, which was associated with an increased efflux of the Pgp substrate rhodamine 123 (Rho123). The chemotherapeutic agent mitomycin C (MMC) was used to study drug-induced trafficking of Pgp. Confocal fluorescence microscopy of single hCMEC/D3-MDR1-EGFP cells revealed that Pgp redistribution from intracellular pools to the cell surface occurred within 2 h of MMC exposure. Pgp-EGFP exhibited a punctuate pattern at the cell surface compatible with concentrated regions of the fusion protein in membrane microdomains, i.e., lipid rafts, which was confirmed by Western blot analysis of biotinylated cell surface proteins in Lubrol-resistant membranes. MMC exposure also increased the functionality of Pgp as assessed in three functional assays with Pgp substrates (Rho123, eFluxx-ID Gold, calcein-AM). However, this increase occurred with some delay after the increased Pgp expression and coincided with the release of Pgp from the Lubrol-resistant membrane complexes. Disrupting rafts by depleting the membrane of cholesterol increased the functionality of Pgp. Our data present the first direct evidence of drug-induced Pgp trafficking at the human BBB and indicate that Pgp has to be released from lipid rafts to gain its full functionality.

## Introduction

The transmembrane drug efflux transporter P-glycoprotein (Pgp; MDR1; ABCB1) contributes to the disposition of a wide variety of drugs of different therapeutic categories due to its extensive tissue distribution and broad substrate specificity [1], [2]. One of its main functions is to protect tissues against endogenous and exogenous toxins by extruding such compounds from the cells, resulting in decreased intracellular drug concentration [3]. Multiple extracellular and intracellular signals regulate the expression and functionality of Pgp, including transcriptional modulation via nuclear receptors, like the pregnane-X receptor, which are involved in drug-induced changes in Pgp expression [4], [5]. In most cells, Pgp is mainly localized in the plasma membrane, but it is also localized in intracellular compartments, such as endoplasmic reticulum, Golgi, endosomes and lysosomes, and cycles between endosomal compartments and the plasma membrane in a microtubular-actin dependent manner [6]. Modulation of trafficking of Pgp from intracellular reservoirs to the cell surface alters post-transcriptional Pgp expression, and may be an effective and rapid way of the cell to respond to potentially toxic compounds by functional membrane insertion of the efflux transporter [7].

Intracellular trafficking of Pgp has been demonstrated for different cell types, particularly liver and cancer cells [6], [8]–[10]. Except for one recent study in rat cerebral microvessel preparations [11] very little is known about the trafficking mechanisms of Pgp and their regulation in the brain capillary endothelial cells that form the blood-brain barrier (BBB). Pgp is an important component of this barrier and is expressed mainly on the apical (luminal) surface of the endothelial cells [5], [12]. In the present study, intracellular trafficking of Pgp was investigated in a human brain capillary endothelial cell line (hCMEC/D3)[13], using a Pgp and enhanced green fluorescent fusion protein (Pgp-EGFP) inducible by doxycycline. To study drug-induced trafficking of Pgp, we used the chemotherapeutic agent mitomycin C (MMC), which has previously been shown to increase membrane-associated Pgp by inducing Pgp trafficking in Madin-Darby canine kidney (MDCK) and rat hepatoma cells [7].

## Methods

### Cell culture conditions

Human cerebral microvascular endothelial cells (hCMEC/D3) were described in detail previously by us [13] and were used for transfection with a doxycycline-inducible MDR1-EGFP fusion plasmid as described below. Both wild type and transfected cells were cultivated in endothelial cell basal medium-2 (EBM-2, Lonza, Cologne, Germany) supplemented with 5% fetal calf serum (PAA Laboratories, Cölbe, Germany), 1% penicillin (100 U/ml), streptomycin (100 µg/ml) (Invitrogen, Karlsruhe, Germany), 1.4 µM hydrocortisone (Sigma-Aldrich, Munich, Germany), 5 µg/ml ascorbic acid (Sigma-Aldrich), 1% lipid concentrate (Invitrogen), 10 mM HEPES (Invitrogen) and 1 ng/ml basic FGF (Sigma-Aldrich). For induction of Pgp-EGFP expression, 1 µg/ml doxycycline (Biochrom, Berlin, Germany) was added to the medium. The dose-dependency of doxcycline's effect on Pgp expression and functionality was studied by exposing the cells to varying concentrations (1 ng/ml, 500 ng/ml, 1 µg/ml) of doxycycline. To control for the effects of doxycycline in the absence of the expression vector, experiments in wild type cells were performed with and without doxycycline. Cells (at passages 30–40) were passaged every 3–4 days on collagen type I- (100 µg/ml) (Invitrogen) coated 100-mm tissue culture plates (Sarstedt, Nuembrecht, Germany) maintained at 37°C and 5% CO_2_.

HEK (human embryonic kidney) 293T cells (DSMZ, Braunschweig, Germany) were cultivated in Dulbecco's modified Eagle's medium containing 4.5 g/l glucose (Sigma-Aldrich), 10% fetal bovine serum (Invitrogen), 100 U/ml penicillin, 100 µg/ml streptomycin (Invitrogen) at 37°C and 5% CO_2_.

### Cloning of MDR1-EGFP-pLox

Due to missing compatible restriction sites in the multiple cloning site (MCS) of the lentiviral expression vector pLOX/TW, which is under control of a tetracycline-responsive promoter [14], the MCS of the vector was extended as a first step. Forward and reverse oligonucleotides were designed containing the sequences of Spe I and Cla I restriction sites and flanked by Sal I and BamH I restriction sites. To anneal the oligonucleotides, equal amounts were mixed, heated to 100°C and cooled down to room temperature. The annealed oligonucleotides and pLOX/TW were restriction digested with Sal I and BamH I. The vector was additionally treated with shrimp alkaline phosphatase (USB, Cleveland, OH, USA) according to the instructions of the manufacturer. After ligation of the oligonucleotide into the pLOX/TW-vector, the vector was electroporated into E. coli SURE bacteria. Subsequently plasmid preparation of pLOX/TW/Spe I-Cla I using JETSTAR Plasmid Kits (Genomed, Löhne, Germany) was performed according to the instructions of the manufacturer and the integrity of the construct was confirmed by sequencing using the ABI Prism 310 capillary sequencer (BigDye Terminator Cycle Sequencing Ready Reaction Mix v. 1.1; Applied Biosystems, Darmstadt, Germany). In a second step MDR1-EGFP was cloned into the lentiviral expression vector pLOX/TW/Spe I-Cla I. To do so, a PCR product containing a MDR1-EGFP fusion construct flanked by Spe I and Cla I restriction sites was generated using a MDR1-linker-EGFP vector (with EGFP placed at the C terminus of MDR1) kindly provided by Piet Borst (The Netherlands Cancer Institute, Amsterdam, The Netherlands) as template. Forward primer (5′-GCA GAC TAG TGC CAC CAT GGA TCT TGA AGG GGA CCG C-3′) comprised a Spe I restriction site and reverse primer (5′-GCT CAT CGA TTT ACT TGT ACA GCT CGT CCA TGC C-3′) a Cla I restriction site (underlined). PCR was performed at 55°C using Pfu Ultra DNA polymerase (Stratagene, LaJolla, CA, USA) according to the instructions of the manufacturer.

After purification (GeneJET™ Gel Extraction Kit; Fermentas, St. Leon-Rot, Germany) the PCR product was restriction digested and ligated into the lentiviral expression vector pLOX/TW/Spe I-Cla I. Amplification of the cloned construct was carried out by electrotransformation into competent E. coli SURE bacteria followed by plasmid preparation using JETSTAR Plasmid Kits (Genomed) according to the instructions of the manufacturer. The integrity of the cloned construct was confirmed by sequencing using the ABI Prism 310 capillary sequencer (BigDye Terminator Cycle Sequencing Ready Reaction Mix v. 1.1; Applied Biosystems).

### Lentivirus production

HEK 293T cells (1×10^7^ cells) were seeded in a 145 cm^2^ petri dish coated with poly-L-lysine and cultivated overnight. Transfection was accomplished by using calcium-phosphate transfection and either 50 µg pUCL-MIK (tetracycline-dependent transactivator) or 50 µg MDR1-EGFP-pLOX plus 12.5 µg pMD.G VSV-G and 50 µg pCMVΔR8.2. Twenty-four hours after transfection, medium was removed and fresh medium containing 10 mM HEPES and 10 mM sodium butyrate was added to the cells. Lentivirus-containing supernatants were harvested and filtered 48 hours after transfection.

### Generation of lentivirally modified hCMEC/D3 cells

2×10^4^ hCMEC/D3 cells were seeded in one well of a 6-well plate. For lentiviral infection of cells, virus containing supernatants (115.4 µl MIK-virus +173.1 µl MDR1-EGFP virus) were diluted in medium to a total volume of 1.2 ml with a final concentration of 8 µg/ml polybrene (Sequabrene, Sigma). Medium was removed from cells and the infection mixtures containing the viruses were added. 24 h after infection, gene expression was induced by adding 1 µg/ml doxycycline (Biochrom) in fresh medium.

### Fluorescence activated cell sorting

EGFP-positive hCMEC/D3-MDR1-EGFP cells were collected by fluorescence-activated cell sorting with a FACS Aria IIu (Becton Dickinson, CA, USA). Dead cells were excluded by using scatter parameters and cell aggregates were excluded by using pulse width.

### Western blot analysis

Human brain endothelial cells (hCMEC/D3, hCMEC/D3-MDR1-EGFP) were lysed in buffer containing 25 mM Tris-HCl, pH 8, 50 mM NaCl, 0.5% (w/v) sodium deoxycholate (DOC), and 0.5% (w/v) Triton X-100 and supplemented with complete protease inhibitor (Roche, Mannheim, Germany). Disruption of cell lysates was performed by drawing up the cell suspension twenty times into a syringe with a small gauge needle (21G) on ice. Protein concentrations in the lysates were determined by using the Pierce BCA Protein Assay kit (Thermo Scientific, Bonn, Germany) according to the manufacturer's instructions. Equal amounts of total protein were separated on 10% SDS-PAGE gels and transferred to PVDF membranes which were blocked overnight in 5% milk in phosphate buffered saline supplemented with Tween-20 (PBST: 137 mM NaCl, 2.7 mM KCl, 4.3 mM Na_2_HPO4, 1.4 mM KH_2_PO4, pH 7.3, 0.05% (w/v) Tween-20) at 4°C. Membranes were incubated with primary antibodies anti-PGP 1∶200 (Signet Laboratories, Dedham, MA, USA) and anti-Actin 1∶100 (Sigma-Aldrich) for 1 h in 2% milk in PBST at room temperature (RT) and washed three times for 10 min in PBS-T. Secondary antibodies anti-mouse-HRP 1∶1000 and anti-rabbit-HRP 1∶1000 (Dako, Hamburg, Germany) were incubated for 1 h in 2% milk in PBST at RT and washed three times for 10 min in PBST. Proteins were detected by enhanced chemiluminescence using SuperSignal West Femto Chemiluminescent Substrate (Thermo Scientific) and the ChemiDoc system (Bio-Rad, Munich, Germany) with QuantityOne software (Bio-Rad) according to the manufacturer's protocol. Relative protein expressions were quantified densitometrically with QuantityOne (Bio-Rad) software and calculated by normalization to the reference signals of actin with GraphPad Prism software (GraphPad, San Diego, CA, USA). For quantification of Pgp expression at the cell surface, Pgp signals were normalized relative to the Coomassie-stained portion of the gel.

### Treatment with mitomycin C

Four days after reaching confluence, cells were stimulated with non-cytotoxic concentrations of MMC (0.1 µM, 1 µM, 10 µM, 50 µM and 100 µM) (Sigma-Aldrich) in Opti-MEM (Invitrogen) at 37°C and 5% CO_2_. If not otherwise indicated, cells were exposed to MMC for 4 h, which was based on previous experiments by Maitra et al. [7] with MDCK and rat hepatoma H4II cells. The effects of MMC exposure were either determined directly after termination of exposure (i.e., at 4 h) or 20 h later, i.e., 24 h after onset of MMC treatment. Control cells were treated only with Opti-MEM (Invitrogen). For 24 hour experiments, Opti-MEM was removed after 4 hours of MMC or control exposure, and cells were treated with fresh culture medium. In preliminary experiments, we also tested the effects of 24 h exposure to MMC (not shown), but since effects were similar to those obtained with 4 h of exposure, all subsequent experiments were performed with the shorter exposure duration.

### Confocal fluorescence microscopic analysis of living cells

hCMEC/D3-MDR1-EGFP cells were plated on collagen type I (100 µg/ml; Invitrogen)-coated 100-mm tissue culture plates (Sarstedt) containing a 42-mm glass plate (H. Sauer Laborbedarf, Reutlingen, Germany). Four days after reaching confluence cells were pretreated with MMC (1 µM) for either 1 or 3 h in Opti-MEM (Invitrogen) at 37°C and 5% CO_2_. Cells were stained for 0.5 h with 5 mM bisbenzimide H (Sigma-Aldrich) in Opti-MEM (Invitrogen) at 37°C and 5% CO_2_. For confocal fluorescence microscopy glass plates with cells were fitted into a PeCon open chamber (PeCon, Erbach, Germany) and MMC (1 µM) in Opti-MEM without phenol red (Invitrogen) was added. Fluorescence images were taken every 3.6 min for the next hour using a Leica SP5 confocal fluorescence microscope with a 63× water objective (Leica Microsystems, Bensheim, Germany) in a climate box (PeCon) at 37°C. Excitation wavelengths of 405 nm (bisbenzimide H) or 481 nm (Pgp-EGFP) were used.

### Cell surface biotinylation and isolation of Lubrol-resistant membranes

hCMEC/D3-MDR1-EGFP cells were plated on collagen type I (100 µg/ml) (Invitrogen) coated 100-mm tissue culture plates (Sarstedt). Four days after reaching confluence the cell surface proteins were isolated. Cells were washed twice with ice- cold PBS and incubated with 10 ml 0.25 mg/ml EZ-Link Sulfo-NHS-SS-Biotin (Thermo Scientific) in PBS for 30 min gently shaking on ice. The reaction was stopped by adding 500 µl Tris (50 mM final concentration, pH 8). Cells were scraped off and collected in 50 ml tubes and centrifuged at 500× g for 5 min at 4°C. The cell pellets were washed with 5 ml Tris-buffered saline (TBS; 25 mM Tris, 0.15 M sodium chloride, pH 7.2) and centrifuged at 500× g for 5 min. After discarding the supernatants, cell pellets were resuspended in lysis buffer (25 mM Tris-HCl, 50 mM NaCl, 0.5% (w/v) DOC and 0.5% (w/v) Triton X-100) supplemented with complete protease inhibitor (Roche). Cell lysates were disrupted by passing the cell suspension twenty times through a syringe with a small gauge needle (21G) and incubated 30 minutes on ice. Samples were centrifuged at 10,000× g for 2 min and the clarified supernatants were mixed with 100 µl Neutravidin Agarose beads (Thermo Scientific) for 1 h with shaking at RT. Thereafter, the beads were washed two times with PBS, 0.5% (w/v) Triton X-100 and 0.05% (w/v) DOC and twice with 500 mM NaCl, 125 mM Tris, 10 mM EDTA and 0.5% (w/v) Triton X-100 (pH 8). Cell surface proteins were eluted from the Neutravidin beads with SDS-PAGE sample buffer under reducing conditions (62.5 mM Tris-HCl, pH 6.8, 1% SDS, 10% (w/v) glycerol and 50 mM dithiothreitol for 1 h with shaking at RT. The eluates were centrifuged for 2 min at 1,000× g and the proteins were analysed by Western blotting as described above. In some experiments Lubrol-detergent extraction of the biotinylated cells was performed by adding 1% (w/v) of Lubrol WX in PBS, and the clarified detergent extracts were centrifuged at 100,000× g for 45 min at 4°C (Beckman Optima LE-80, SW-55Ti rotor). Lubrol-resistant membranes (DRMs) retained in the pellet were resuspended in PBS containing 0.5% (w/v) DOC and 0.5% (w/v) Triton X-100 and the protease inhibitor cocktail. Treatment of the resuspended DRMs as well as the supernatants with Neutravidin-Agarose beads and analysis by Western blotting were performed as described above.

### Rhodamine 123 uptake assay

Uptake assays with the cell-permeant, cationic, green-fluorescent Pgp substrate rhodamine 123 (Rho123; Sigma-Aldrich) were performed to evaluate Pgp transport function [13], [15], [16]. In our hands this assay was more sensitive and less variable to determine drug-induced alterations in Pgp functionality than several other uptake assays with Pgp substrates, including vinblastine and digoxin, which we previously used [17]. For the Rho123 uptake assay, cells were grown on collagen type I (100 µg/ml) (Invitrogen) coated 6-well plates (Greiner, Frickenhausen, Germany). Four days after reaching confluence cells were stimulated with MMC as described above. At different times after MMC exposure, the cells were incubated with 10 µM Rho123 (Sigma-Aldrich) in Opti-MEM (Invitrogen) shaking for 1 h at 37°C and 5% CO_2_. Cells were washed twice with PBS and scraped in 500 µl ice cold PBS and collected in 1.5 ml tubes, which were centrifuged eight minutes at 300× g at 4°C. The cell pellet was resuspended in 200 µl lysis buffer (25 mM Tris-HCl, 50 mM NaCl, 0.5% (w/v) DOC and 0.5% (w/v) Triton X-100). Protein determination and Western blots were performed as described above. Fluorescence was measured with the FLUOstar OPTIMA (BMG Labtech, Ortenberg, Germany) and was calculated as absolute fluorescence in the cell lysate per mg of protein. The extent of Rho123 uptake varied between different cell preparations, so that concurrent controls were used in all experiments.

In some experiments, the Pgp inhibitor tariquidar (0.5 µM) was added to the cells 1 h before Rho123 and during the 1 h of incubation with Rho123 in order to determine whether the alterations in Rho123 uptake induced by transfection with Pgp-EGFP or exposure to MMC could be counteracted by inhibition of Pgp.

### Rhodamine 123 efflux assay

For using the Rho 123 assay in the efflux configuration [18], cells were preloaded with 10 µM Rho123 (Sigma Aldrich) in Opti-MEM (Invitrogen) for 15 min on ice. After cells were washed twice in PBS and medium was changed to Opti-MEM (Invitrogen) without Rho123, the cells were placed in the incubator (37°C and 5% CO_2_) and the decay of intracellular florescence was measured after 15, 30 and 60 min in the absence or presence of the Pgp inhibitor tariquidar (0.5 µM). To control Rho123 preloading, cells were directly harvested after preincubation. Fluorescence was measured as described above and was calculated as fluorescence efflux per min.

### eFluxx-ID Gold uptake assay

To confirm the findings of the Rho123 assay with a second method, we used the fluorescent probe eFluxx-ID Gold, a recently presented xanthene-based small molecule dye that, in combination with a Pgp inhibitor, can be used to monitor Pgp functionality in live cells [19], [20]. hCMEC/D3-MDR1-EGFP (doxycycline-on) cells were trypsinized and incubated for 30 min at 37°C in phenol red free Opti-MEM medium (Invitrogen) with eFluxx-ID Gold (ENZO Life Sciences, Lörrach, Germany) according to the manufacturer's protocol. Cells were analyzed by flow cytometry (FACSCanto; BD Biosciences, Heidelberg, Germany). Each flow cytometry analysis consisted of a record of 100,000 cells. The uptake of eFluxx-ID Gold of EGFP-positive cells was measured in the FL2 (PE) and FL1 (FITC) channel. Dead cells were excluded by using scatter parameters. In parallel experiments, tariquidar (0.5 µM) was added to the cells 1 h before eFluxx-ID Gold and during the incubation with eFluxx-ID Gold in order to determine whether the alterations in eFluxx-ID Gold uptake induced by transfection with Pgp-EGFP or exposure to MMC could be counteracted by inhibition of Pgp.

### Calcein-AM extrusion assay

To confirm the findings of the uptake assays (Rho123 and eFluxx-ID Gold) with a third method, we performed the calcein-AM extrusion assay [21]. The lipophilic nonfluorescent acetoxymethyl (AM) ester of calcein diffuses into cells where it is hydrolyzed to calcein, a fluorescent organic anion. Because calcein-AM is effluxed by Pgp, the intracelluar accumulation of calcein is measured as an indirect (and inverse) measure of calcein AM efflux [21]. For the present experiments, we performed the assay as described by Bauer et al. [22]. Following exposure to MMC, hCMEC/D3-MDR1-EGFP (doxycycline-on) cells were washed two times with PBS and were subsequently incubated with 2 µM calcein-AM (Sigma-Aldrich) for 15 min at 37°C. Cells were harvested and further analysed as described in Rho123 uptake assay section. In further experiments for calcein-AM kinetic studies, which were performed essentially as described by Bauer et al. [22], cells were seeded on 96-well plates. Cells were preloaded with calcein-AM (0.25 µM, 0.5 µM, 1 µM or 2 µM) and transferred into a FLX 800 microplate reader (Bio Tek, Bad Friedrichshall, Germany) for 1 h at 37°C where fluorescence was measured every 30 seconds and was calculated as absolute fluorescence per second per mg of protein. In parallel experiments, tariquidar (0.5 µM) was added to the cells 1 h before calcein-AM and during the incubation with calcein-AM in order to determine whether the alterations in calcein accumulation induced by transfection with Pgp-EGFP or exposure to MMC could be counteracted by inhibition of Pgp.

### Treatment with cyclodextrin

For depletion of cell membrane cholesterol, methyl-β-cyclodextrin (MβCD, Sigma-Aldrich) was used [23]. MβCD (Sigma-Aldrich) was dissolved in Opti-MEM medium (Invitrogen) at a final concentration of 10 mM. hCMEC/D3-MDR1-EGFP cells were treated for two hours with 10 mM MβCD. Cells were washed twice with PBS and rhodamine 123 uptake assays were performed as described above.

### Flow cytometric analysis

hCMEC/D3 wild type, hCMEC/D3-MDR1-EGFP (doxycycline-on) and hCMEC/D3-MDR1-EGFP (doxycycline-off) cells were trypsinized and flow cytometric analysis were performed on a FACSCanto (BD Biosciences) using forward (FSC) and side (SSC) scattering parameters.

### Statistics

Drug effects were compared with individual controls, using either Student's t-test or, when data were not normally distributed, the U-test of Mann and Whitney, using PRISM 5 software (GraphPad Software Inc., La Jolla, CA, USA). For analysis of differences between several groups in the same experiment, analysis of variance (ANOVA) followed by Dunnett's or Bonferroni's Multiple Comparison Tests were used. Tests used were two-sided and a P<0.05 was considered significant.

## Results

### Generation of conditional doxycycline-dependent Pgp-EGFP expressing hCMEC/D3-MDR1-EGFP cells

To investigate Pgp trafficking, we generated conditional doxycycline-dependent Pgp-EGFP expressing hCMEC/D3-MDR1-EGFP cells with a lentiviral expression system. As a template for cloning MDR1-EGFP in the lentiviral expression vector (pLOX/TW/Spe I-Cla I) we used a MDR1-linker-EGFP construct that was previously generated by Piet Borst (see experimental procedures) and, to our knowledge, has never been used before in any published study. In the MDR1 sequence of this construct we identified three of the most common single nucleotide polymorphisms, T1236C (Gly412Gly), T2677G (Ser893Ala) and T3435C (Ile1145Ile), which have been described in MDR1 of humans [24]. To produce MDR1-EGFP-containing lentiviral particles, HEK293T cells were transfected with MDR1-EGFP-pLOX/TW/Spe I-Cla I, pMD.G VSV-G and pCMVΔR8.2. These lentiviral particles were then used to infect hCMEC/D3-cells. Doxycycline-dependent expression of MDR1-EGFP was mediated by coinfection of hCMEC/D3 cells with MIK-lentivirus encoding the tet-transactivator. The successful infection was detected by EGFP fluorescence and Western blot analysis of the Pgp-EGFP fusion protein (data not shown). Subsequently, EGFP positive hCMEC/D3 cells (6.4%) were isolated by fluorescence-activated cell sorting.

### Characterization of conditional doxycycline-dependent Pgp-EGFP expressing hCMEC/D3-MDR1-EGFP cells

**Figure 1:**
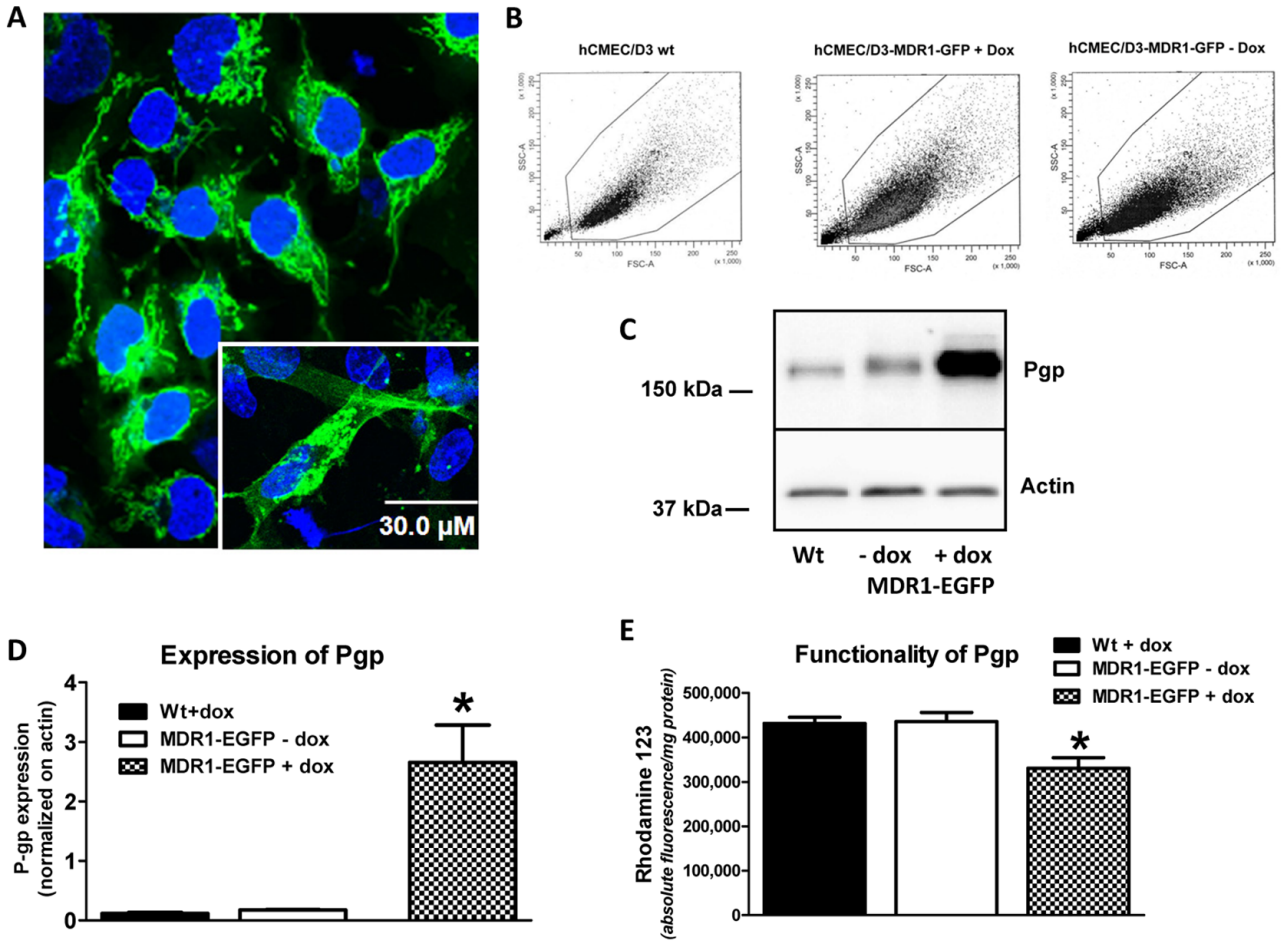
Conditional doxycycline-dependent Pgp-EGFP-expressing hCMEC/D3-MDR1-EGFP cells. (A) Representative images showing intracellular localization of Pgp-EGFP fusion protein in hCMEC/D3-MDR1-EGFP (doxycycline-on) cells. Cell nuclei of living cells were stained with bisbenzimide H (blue) (scale bar = 30 µm). (B) Flow cytometric analysis of relative cell size and granularity of hCMEC/D3 wild type, hCMEC/D3-MDR1-EGFP (doxycycline-on) and hCMEC/D3-MDR1-EGFP (doxycycline-off) cells, indicating the lack of any obvious differences between the three conditions; furthermore, as indicated by comparable extent of cell debris, the vitality of the cells does not differ. Along the X-axis is the FSC(Forward SCatter) parameter, while the Y-axis shows the SSC(Side SCatter) parameter. (C,D) Significant Pgp-EGFP fusion protein induction in hCMEC/D3-MDR1-EGFP cells by doxycycline (1 µg/ml) was analyzed by Western blot. Control whole cell lysates of hCMEC/D3 wild type (Wt) and hCMEC/D3-MDR1-EGFP (doxycycline-off/on) cells were used. In C, one representative Western blot of three is shown. Bands were analysed densitometrically and Pgp signals were normalized on actin (D). (E) Induction of Pgp-EGFP by doxycycline (1 µg/ml) significantly decreases Rho123 accumulation in hCMEC/D3-MDR1-EGFP cells confirming that Pgp-EGFP fusion protein is functional. Data in (D) and (E) are shown as mean ± SEM of three experiments. Asterisks denote values that were significantly different from corresponding controls (P<0.05). In all experiments with wild type cells, doxycycline was added for control in order to exclude that doxycycline induces Pgp expression and function also in the absence of an expression vector (see also Figure 2A).

Confocal fluorescence microscopic analysis of sorted hCMEC/D3-MDR1-EGFP (doxycycline-on) cells revealed a predominant intracellular localization of the Pgp-EGFP fusion protein (Figure 1A). hCMEC/D3-MDR1-EGFP cells are therefore well suited to analyse membrane-associated trafficking from intracellular pools. The phenotype (size, granularity, vitality) of the transfected cells (with or without doxycycline) did not differ from wild type when analyzed by flow cytometry (Figure 1B). To assess functionality of Pgp-EGFP inducible doxycycline-on/-off-system we compared Pgp expression of non-induced Pgp-EGFP (doxycycline-off) and induced Pgp-EGFP (doxycycline-on) hCMEC/D3-MDR1-EGFP cells versus hCMEC/D3 wild type cells by Western blot analysis (Figure 1C). hCMEC/D3 wild type cells expressed a 170-kDa protein band corresponding to endogenous Pgp whereas non-induced hCMEC/D3-MDR1-EGFP cells expressed an approximately 200-kDa protein band corresponding to the predicted size of the chimeric MDR1-EGFP (Figure 1C). In the induced cells, the 200 kDa band was too intense to visualize any endogenous Pgp. When normalized to actin, the expression level of Pgp in hCMEC/D3 wild type cells did not differ significantly from that of the Pgp-EGFP fusion protein in hCMEC/D3-MDR1-EGFP (doxycycline-off) cells (Figure 1D). Furthermore, the functionality assessed by efflux of Rho123 was very similar (Figure 1E). In the presence of doxycycline, hCMEC/D3-MDR1-EGFP cells exhibited an about 15-fold increase in Pgp-EGFP fusion protein expression (Figure 1D) and significantly decreased Rho123 accumulation in comparison to non-induced hCMEC/D3-MDR1-EGFP (Figure 1E). Compared to hCMEC/D3-MDR1-EGFP without doxycycline, the decrease in Rho123 accumulation in doxycycline-induced hCMEC/D3-MDR1-EGFP cells was only 24.1% on average (Figure 1E), substantiating the findings from confocal fluorescence microscopic analysis of these cells (Figure 1A) that most of the Pgp-EGFP fusion protein was localized intracellularly and thus not functionally expressed at the cell membrane. We repeated the experiment illustrated in Figure 1E several times (for example see Figure S1) and always determined a significant decrease in Rho123 accumulation in the doxycycline-induced cells, but the extent of this decrease varied between different cell preparations between 24.1 and 72.0% (mean 46.2%; 5 experiments with 3 samples per experiment).

In addition to performing the Rho123 assay as an accumulation assay as shown in Figure 1E, we also used an efflux configuration with preloading the cells with Rho123 at 4°C and then measuring the decay of intracellular fluorescence at 37°C, thus determining efflux rates in the presence and absence of tariquidar (Figure S2). This assay demonstrated the enormous efflux capacity of the doxycycline-induced hCMEC/D3-MDR1-EGFP cells. Pgp inhibition by tariquidar significantly reduced the Rho123 efflux, but inhibition was only partial under these conditions (Figure S2), which may indicate that either the concentration of tariquidar used (0.5 µM) was not sufficient to completely block the Pgp-mediated efflux of Rho123 or that other transporters were involved in this efflux.

To study the dose-dependence of induction of Pgp expression and functionality by doxycycline, hCMEC/D3-MDR1-EGFP cells were exposed to different concentrations of doxycyline, ranging from 1 ng/ml to 1 µg/ml. Doxycycline dose-dependently increased both Pgp expression and its functionality (as measured by the Rho123 assay) over this concentration range (Figure S1). In all subsequent experiments, a concentration of 1 µg/ml was used, because this resulted in the most intense labeling of the Pgp-EGFP fusion protein in confocal fluorescence microscopic analysis of hCMEC/D3-MDR1-EGFP cells.

**Figure 2:**
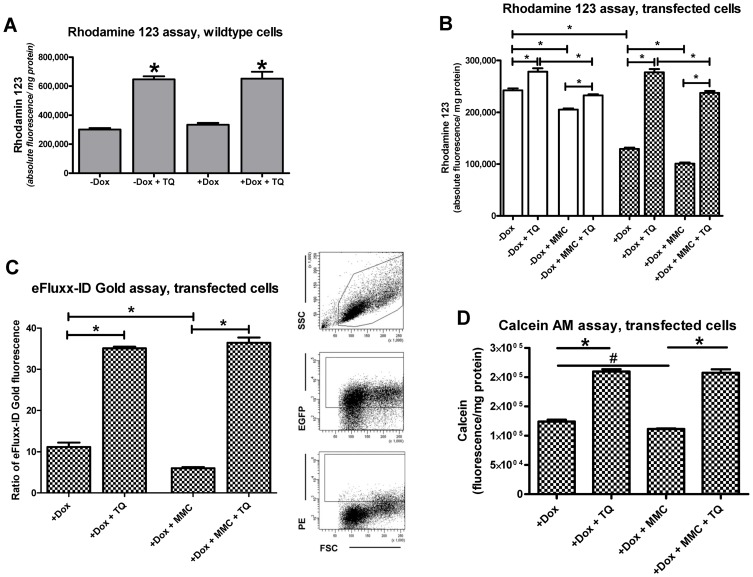
Effects of the Pgp inhibitor tariquidar (TQ; 0.5 µM) in three functional assays, in which alterations in Pgp efflux are indirectly measured by determining intracellular concentrations of Pgp substrates (Rho123, eFluxx-ID Gold) or their metabolite (calcein AM). Data are shown as mean ± SEM of three experiments; all experiments with one uptake assay were performed together to avoid inter-experiment variation. In experiments with mitomycin C (MMC) (B,C,D), experiments were performed 20 h after a 4-h exposure to 1 µM MMC. Significant differences between treatments are indicated by asterisk (P<0.01) except for D, in which significance level of the effect of MMC was P = 0.0188 (indicated by rhomb). (A) shows data from the Rho123 uptake assay in nontransfected (wild type) hCMEC/D3 cells in the absence or presence of doxycycline (Dox). Doxycycline (1 µg/ml) did not alter the functionality of Pgp. Tariquidar significantly increased the uptake of Rho123 in wild type cells both in the absence and presence of doxycycline to the same extent. Much higher concentrations (100 µg/ml) of doxycycline have been shown to increase Pgp expression and functionality in MCF-7 breast carcinoma cells [37]. (B) shows data from the Rho123 uptake assay in transfected hCMEC/D3-MDR1-EGFP cells in the absence and presence of doxycycline. In the absence of doxycycline (open columns), tariquidar significantly increased Rho123 accumulation (i.e., decreased Rho123 efflux) by only about 15%. In the presence of doxycycline, accumulation of Rho123 was only about 50% of that under doxycycline-off conditions, which was completely counteracted by tariquidar. When experiments were performed 20 h after a 4-h exposure to 1 µM MMC, Rho123 accumulation was significantly reduced by 15% under doxycycline-off and 22% under doxycycline-on conditions. Tariquidar counteracted the enhanced functionality of Pgp in response to MMC exposure. However, the tariquidar-induced increase in Rho123 accumulation in MMC-exposed cells remained significantly below the increase seen in the absence of MMC, which was seen both under doxycycline-on and -off conditions. (C) shows data from intracellular accumulation of eFluxx-ID Gold in transfected hCMEC/D3-MDR1-EGFP cells in the presence of doxycycline. In this assay, by using scatter parameters and FL1 (FITC) channel, only viable and EGFP-positive cells were analyzed for intracellular accumulation of the fluorescent Pgp probe. Following MMC exposure, eFluxx-ID Gold accumulation was significantly reduced by 46% under doxycycline-on conditions. Tariquidar completely counteracted the enhanced functionality of Pgp in response to MMC exposure. (D) shows data from intracellular accumulation of calcein in transfected hCMEC/D3-MDR1-EGFP cells in the presence of doxycycline. Following MMC exposure, calcein accumulation was significantly reduced by 10% under doxycycline-on conditions. Tariquidar completely counteracted the enhanced functionality of Pgp in response to MMC exposure.

Exposure of wild type cells to doxycycline did not induce the expression or functionality of Pgp (Figure 2A). In doxycycline-induced hCMEC/D3-MDR1-EGFP cells, the Pgp inhibitor tariquidar counteracted the increased efflux of Rho123 in both versions of this assay (Figure S2 and Figure 2B). In addition to Rho123, we also used the eFluxx-ID Gold and calcein-AM assays to demonstrate functionality of Pgp in the MDR1-EGFP transfected cells (Figure 2C,D). In all assays, tariquidar significantly counteracted the efflux of the Pgp substrates (Figure 2B,C,D).

In the calcein AM extrusion assay, we also determined the transport rate of calcein AM efflux (by measuring intracellular calcein) in doxycycline-induced hCMEC/D3-MDR1-EGFP cells at different concentrations of the Pgp substrate (Figure S3). Average transport rate (in fluorescence of intracellular calcein per sec per mg protein) linearily increased with increasing concentrations of calcein AM and was 3.9 at 0.25 µM calcein AM, 7.2 (0.5 µM), 15.0 (1 µM), and 37.0 (2 µM), respectively (r = 0.9904; P = 0.0048 when analyzed by linear regression analysis). Transport was inhibited by tariquidar at all concentrations of calcein AM in this assay (Figure S3).

### Mitomycin C-induced trafficking of Pgp-EGFP fusion protein

**Figure 3:**
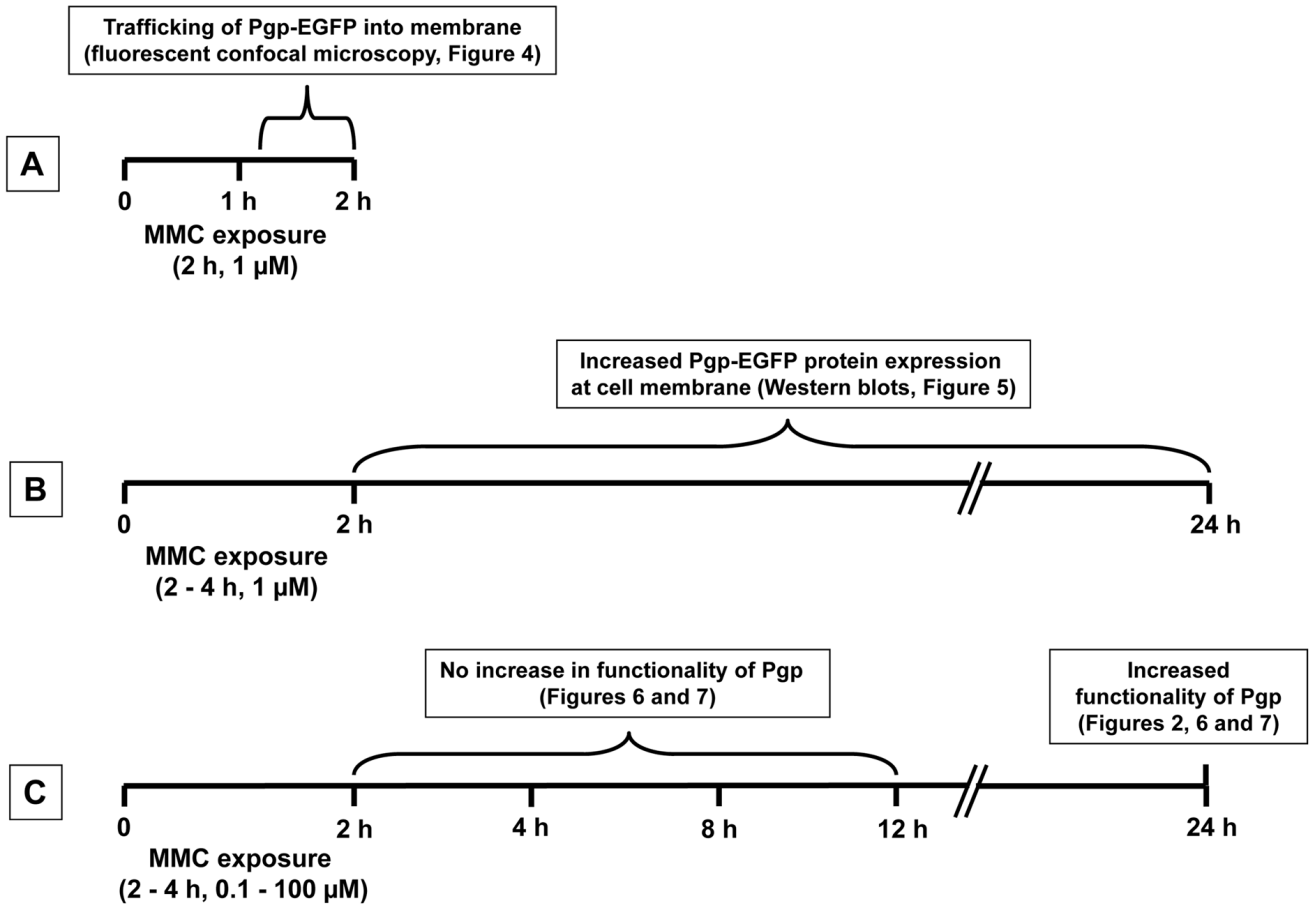
Schematic illustration of the three experimental strategies used for determining whether exposure to mitomycin C (MMC) induces trafficking of Pgp to the plasma membrane in hCMEC/D3-Pgp-EGFP cells.

As summarized in Figure 3, three experimental strategies were used to determine whether MMC exposure induces Pgp trafficking from intracellular compartments to the plasma membrane in human brain endothelial cells, hCMEC/D3-MDR1-EGFP (doxycycline-off and doxycycline-on), and whether this trafficking increases the functionality of the transporter.

### Confocal fluorescence microscopic analysis of Pgp trafficking

**Figure 4:**
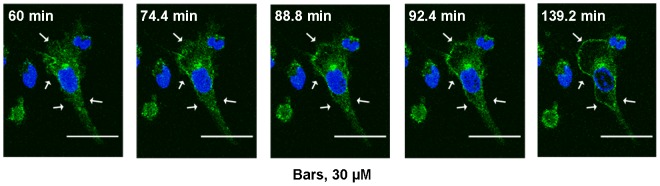
Mitomycin C induced Pgp-EGFP trafficking in hCMEC/D3-MDR1-EGFP (doxycycline-on) cells. A representative experiment is shown. hCMEC/D3-MDR1-EGFP (doxycycline-on) cells were pretreated with 1 µM MMC for 1 h and cell nuclei of living cells were stained with bisbenzimide H (blue) (scale bars = 30 µm). After 1 h of MMC exposure, fluorescence images were taken every 3.6 min for another 1 h in the presence of MMC. Trafficking events of Pgp-EGFP fusion protein are labeled (arrows). Within 15 min after onset of confocal microscopic analysis (i.e., about 75 min after onset of MMC exposure), we observed that Pgp-EGFP started to traffick from intracellular compartments to the membrane. See Movie S1 illustrating the whole period between 60 min and 139.2 min in fast motion.

First, we used confocal fluorescence microscopic analysis of single living human endothelial cells to follow the trafficking of the Pgp-EGFP chimeras (Figure 4). When cells were exposed for 4 h to MMC and observed by confocal microscopy in the last hour of the exposure period, no changes in the intracellular localization of Pgp-EGFP could be detected (not shown). To examine the possibility that this is due to a rapid trafficking of Pgp-EGFP after administration of MMC and does not reflect an intracellular block of the chimeric protein, we shortened MMC treatment to 2 h and observed the cells during the second hour of exposure (Figure 3A). Under these conditions, the MDR1-EGFP fusion protein trafficked from intracellular pools to the cell surface (Figure 4 and Movie S1). The first detectable trafficking event required approximately 15 min. Following a halt of Pgp membrane trafficking of approximately 20 minutes, a second trafficking event could be observed. This required approximately 50 minutes in which Pgp-EGFP became distributed at the cell surface membrane. Strikingly, Pgp-EGFP exhibited a punctuate pattern at the cell surface compatible with concentrated regions of the fusion protein in particular membrane patches, presumably membrane microdomains (Figure 4 and Movie S1). When using the same time frame in untreated control cells, we only observed intracellular trafficking, but no trafficking to the cell membrane as observed with MMC (Movie S2). During the preparation of our manuscript, Huber et al. [20] demonstrated by using spectral position determination microscopy (SPDM) that Pgp clusters in the membrane of hCMEC/D3 cells, thus supporting our findings. Nevertheless, trafficking of Pgp was not addressed in their study.

### Western blot analyses of Pgp trafficking

**Figure 5:**
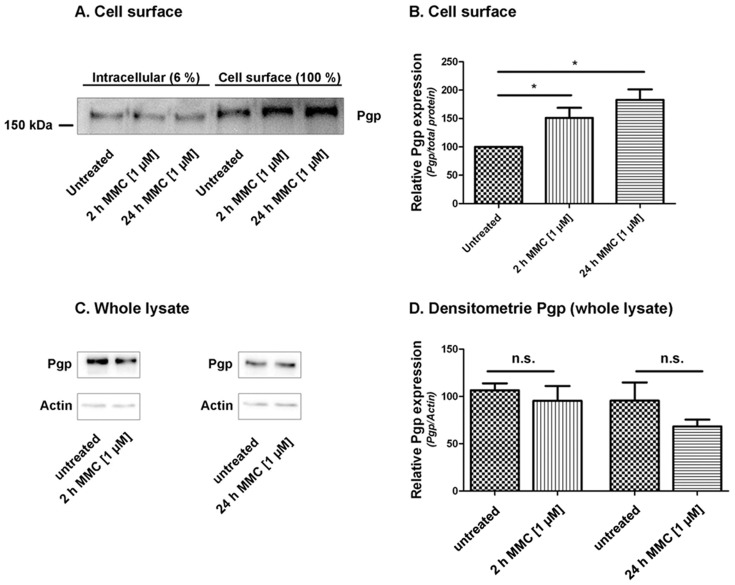
Treatment with mitomycin C (MMC) increases Pgp-EGFP fusion protein at the cell surface. (A) hCMEC/D3-MDR1-EGFP (doxycycline-on) cells were treated with MMC (1 µM) for 2 or 4 h and analyzed at the end of the 2 h-exposure period or 20 h after the 4 h-exposure period. Western blot analyses of cell surface proteins isolated via EZ-Link Sulfo-NHS-SS-Biotin-Neutravidin assay revealed an obvious increase of Pgp-EGFP abundance at the cell surface after MMC exposure, whereas no effect on intracellular Pgp was seen. One representative result of six independent experiments is shown. In (B), Pgp-EGFP bands of the cell surface were analyzed densitometrically and Pgp signals were normalized relative to the coomassie-stained portion of the gel. Data variability is shown as ± SEM of six experiments; significant differences of treated vs. untreated samples are indicated by asterisk (P<0.05). (C, D) No significant induction of Pgp-EGFP fusion protein was measured in whole cell lysates at the same exposure conditions used in (A). Respective P values in D were 0.5502 (2 h MMC) and 0.2534 (24 h MMC). Pgp-EGFP bands were analyzed densitometrically and Pgp signals were normalized on actin. Data variability is shown as ± SEM of three experiments.

Next, we wanted to corroborate biochemically our findings of a cell surface expression of Pgp-EGFP after MMC treatment by Western blot analyses. As shown in Figure 5A, Pgp-EGFP fusion protein was significantly increased at the plasma membrane upon MMC treatment, whereas the total amount of Pgp-EGFP fusion protein was not significantly changed in whole cell lysates of MMC treated hCMEC/D3-MDR1-EGFP cells (Figure 5C,D). Following 2 h of MMC (1 µM) exposure, the expression level of Pgp-EGFP fusion protein at the plasma membrane increased by approximately 50% at the end of exposure (Figure 5B). Twenty h after a 4-h exposure with MMC, the average increase reached 80% (Figure 5B).

### Functional analysis of Pgp trafficking by the rhodamine 123 assay

**Figure 6:**
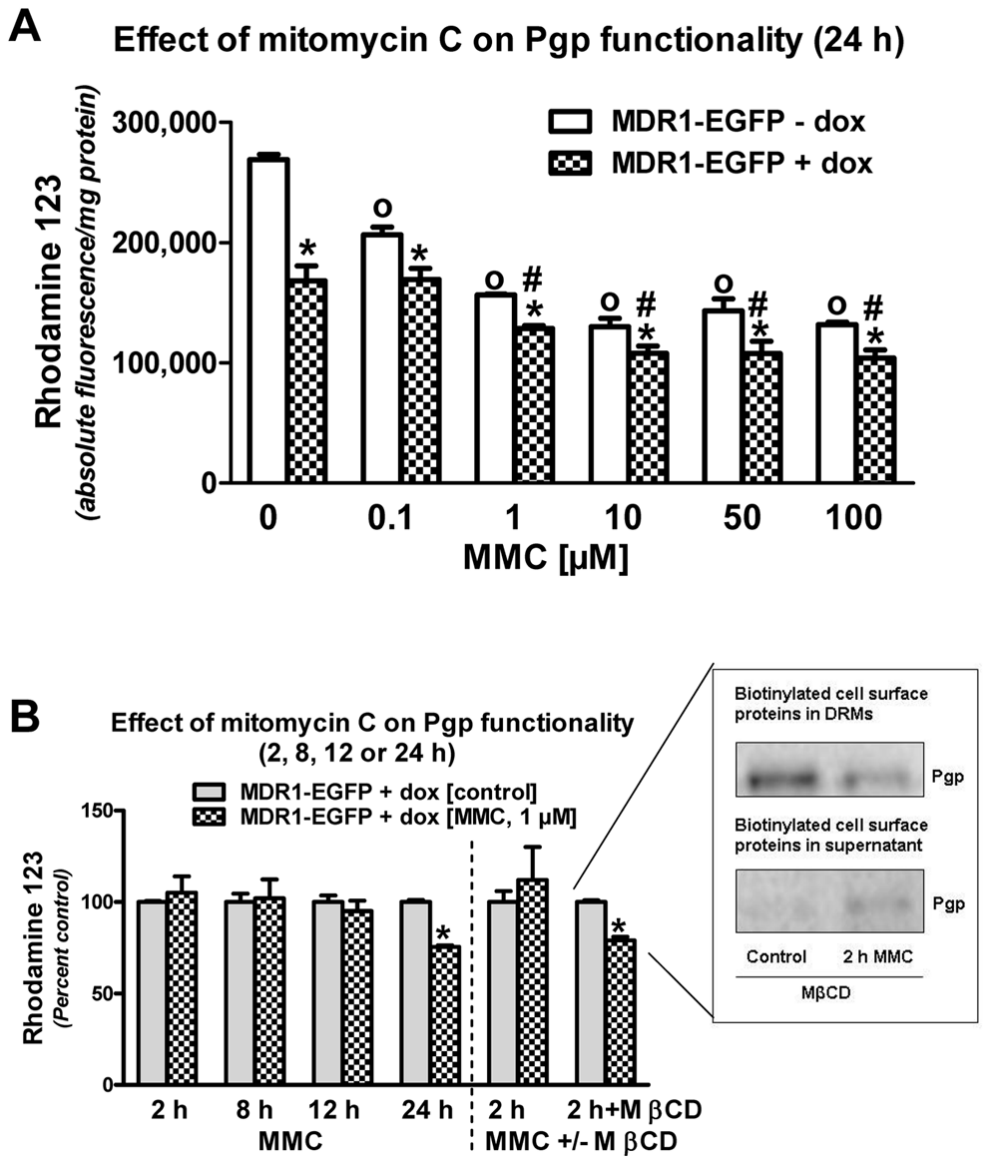
Effect of mitomycin C (MMC) on Pgp-EGFP functionality studied with the Rho123 uptake assay. MMC significantly increased the functionality of Pgp-EGFP fusion protein. “A”: hCMEC/D3-Pgp-EGFP (doxycycline-off/-on) cells were treated with MMC in Opti-MEM medium (0.1 µM, 1 µM, 10 µM, 50 µM and 100 µM) for 4 hours after which Opti-MEM medium was replaced by complete medium again. Control cells were treated with Opti-MEM medium. After 23 h the cells were incubated with 10 µM Rho123 for 1 h and intracellular accumulation was measured, resulting in a dose-dependent increase in Pgp-EGFP functionality. Experiments were performed in triplicates and values are shown as mean ± SEM. Significant effects of MMC during doxycycline-off conditions are indicated by circles, while significant effects of MMC during doxycycline-on conditions are indicated by rhombs (P<0.05). Significant differences between doxycycline-on and –off conditions are indicated by asterisk (P<0.05). “B” shows data (shown in percent control) in which rhodamine uptake was measured directly after 2 h of MMC (1 µM) exposure or at 4, 8, and 20 hrs following 4 h of MMC (1 µM) exposure in hCMEC/D3-Pgp-EGFP (doxycyline-on) cells, indicating a lack of MMC exposure on Pgp function in the 2, 8 and 12, but not the 24 h experiment. The 24 h experiment was also performed with 10 µM MMC, resulting in similar results (not illustrated). However, when lipid rafts were disrupted by MβCD, MMC (1 µM) exposure for 2 h significantly increased the functionality of Pgp-EGFP fusion protein, which is illustrated by the right columns in “B”. As shown in the inset, this was correlated with a decrease of Pgp in the DRM fraction and an increase of Pgp in the detergent soluble fraction. Significant differences between controls and MMC-treated cells are indicated by asterisk (P<0.05).

In order to determine whether the MMC-induced trafficking of the Pgp-EGFP fusion protein increased Pgp-mediated drug efflux, the efflux of the Pgp substrate Rho123 was studied, under both doxycycline-off and –on conditions (Figure 3C). As shown in Figure 6A, a significant decrease in Rho123 accumulation was observed in hCMEC/D3 (doxycycline-off) cells stimulated with MMC (0.1 µM, 1 µM, 10 µM, 50 µM and 100 µM), when the experiment was performed 20 h after 4 h MMC exposure, thus demonstrating that MMC increased the functionality of Pgp. The maximum effect of MMC (∼50% vs. control) was determined at concentrations of 10-100 µM MMC. In doxycycline-induced hCMEC/D3-MDR1-EGFP cells, rhodamine accumulation was significantly decreased (by 37%) already in the absence of MMC, which was further enhanced by MMC at concentrations of 1-100 µM (Figure 6A). The maximum effect (−38% vs. control) was observed at concentrations of 10–100 µM MMC. Overall, the doxycycline-induced cells seemed to be less sensitive to MMC than the non-induced cells (but see Figure 2). When the experiment with 1 µM MMC was repeated several times in doxycycline-induced hCMEC/D3-MDR1-EGFP cells, the effect size of the decrease in Rho123 uptake 20 h after 4 h of MMC exposure was quite consistent with −22% (Figure 2B), −23.7% (Figure 6A) and −24.4% (Figure 6B), respectively. However, such an effect was not observed at earlier time points following MMC exposure (Figure 6B), indicating a temporal disconnect between the Pgp trafficking and the time to reach measurable alterations in Pgp function.

The increased efflux of Rho123 in response to MMC exposure was significantly reduced by tariquidar under both doxycycline-off and –on conditions (Figure 2B). However, as shown in Figure 2B, tariquidar did not completely counteract the effect of MMC, which may indicate a non-specific effect of MMC on Rho123 distribution. We therefore used two additional assays (eFluxx-ID, calcein AM) to study the effects of MMC on Pgp functionality.

### Functional analysis of Pgp trafficking by the eFluxx-ID Gold assay

**Figure 7:**
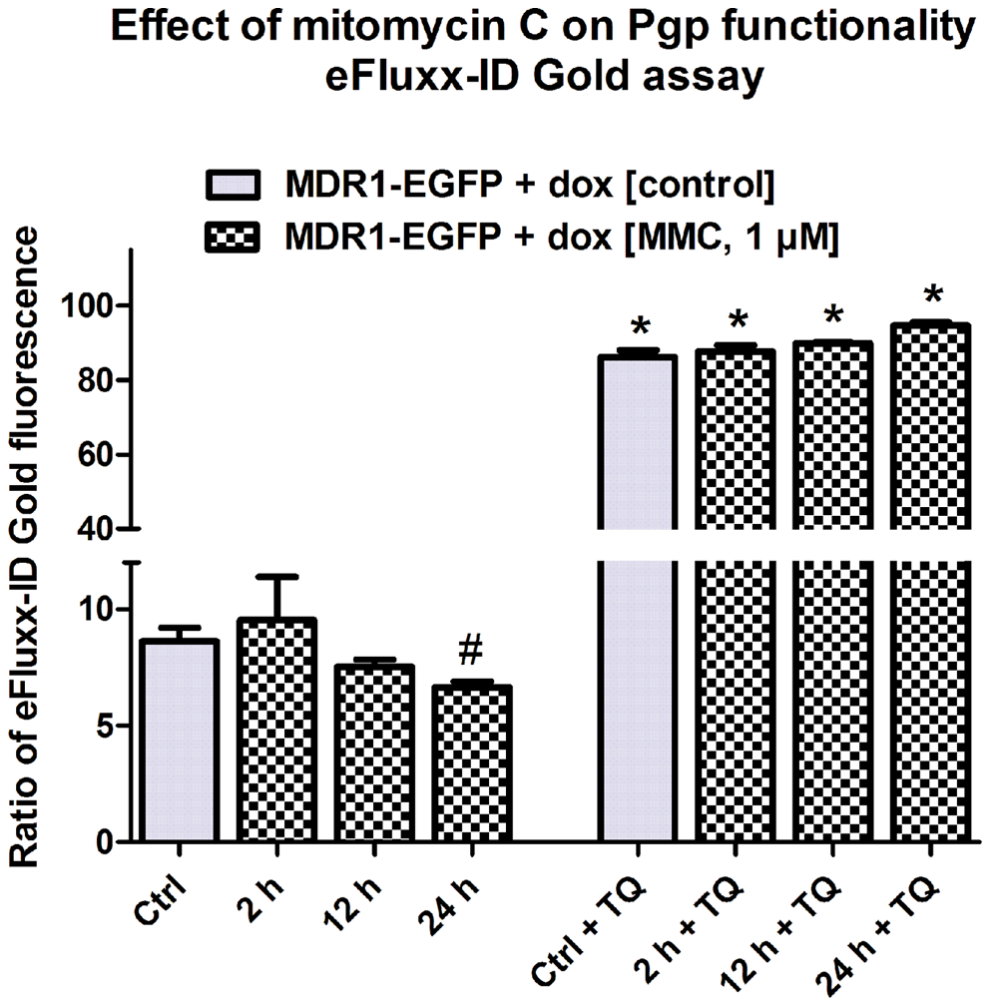
Effect of mitomycin C (MMC) on Pgp-EGFP functionality studied with the eFluxx-ID Gold assay in the absence and presence of the Pgp inhibitor tariquidar (TQ; 0.5 µM). The figure shows data in which eFluxx-ID Gold uptake was measured directly after 2 h of MMC (1 µM) exposure or at 8 and 20 hrs following 4 h of MMC (1 µM) exposure in hCMEC/D3-Pgp-EGFP (doxycyline-on) cells, indicating a lack of MMC exposure on Pgp function in the 2 and 12 h, but not the 24 h experiment. Experiments were performed in triplicates and values are shown as mean ± SEM. Significant differences between control or MMC exposure with tariquidar vs. exposure without tariquidar are indicated by asterisk (P<0.001) while significant difference between MMC exposure (without tariquidar) and control is indicated by rhomb (P = 0.0353).

The eFluxx-ID Gold assay was performed to analyze MMC dependent Pgp activity of only viable and EGFP-positive cells (Figure 2C). Intracellular accumulation of eFluxx-ID Gold was significantly reduced by 46% upon MMC treatment (1 µM) of hCMEC/D3-MDR1-EGFP cells under doxycycline-on conditions (Figure 2C). In the presence of tariquidar, the Pgp activity of MMC treated cells was completely antagonized in comparison to untreated cells (Figure 2C), thus arguing against any non-specific effects of MMC as indicated by the Rho123 data shown in Figure 2B. Instead, when comparing the MMC data from the Rho123 and eFluxx-ID Gold assays, the Rho123 assay seemed to underestimate the effect of MMC on Pgp functionality. To further examine whether the data on the temporal effects of MMC exposure on Pgp functionality obtained with the Rho123 assay (Figure 6B) correctly indicated a temporal disconnect between Pgp trafficking and the time to reach measurable alterations in Pgp function, these experiments were repeated with the eFluxx-ID Gold assay. As shown in Figure 7, this experiment confirmed the data with the Rho123 assay that a significant increase in Pgp functionality was only observed 24 h after onset of a 4 h MMC exposure. Again, tariquidar completely counteracted the MMC-induced increase in Pgp functionality in the eFluxx-ID Gold assay (Figure 7).

### Functional analysis of Pgp trafficking by the calcein AM extrusion assay

In the calcein AM extrusion assay, we studied the effect of MMC (1 µM) following 20 h of 4 h exposure with MMC (Figure 2D). MMC exposure significantly decreased intracellular calcein accumulation, indicating an increased Pgp functionality, which could be completely counteracted by tariquidar, thus confirming the results from the two other accumulation assays. However, the effect size resulting from MMC exposure (∼10%) in the calcein-AM assay was much smaller compared to the other two assays. In an additional experiment illustrated in Figure S3, we determined the effect of MMC on the transport rate in the calcein AM extrusion assay. Furthermore, we examined whether the concentration of calcein AM had an influence of the effect of MMC. At all concentrations of calcein AM studied, MMC caused a small (7–11%) but statistically significant decrease in intracellular calcein, indicating enhanced efflux of calcein AM. The effect of MMC was completely counteracted by tariquidar in all experiments shown in Figure S3.

### Role of lipid rafts in the temporal disconnect between Pgp trafficking and increased Pgp function

These data with three different accumulation assays indicated that MMC exposure increased the functional expression of Pgp-EGFP fusion protein when the assays were performed 20 h after 4 h of MMC exposure. However, no significant decrease in Rho123 or eFluxx-ID Gold accumulation was observed when the experiments were performed directly after 2 h of MMC exposure or at 4 and 8 hrs following 4 h of MMC exposure (Figure 6B, Figure 7).

**Figure 8:**
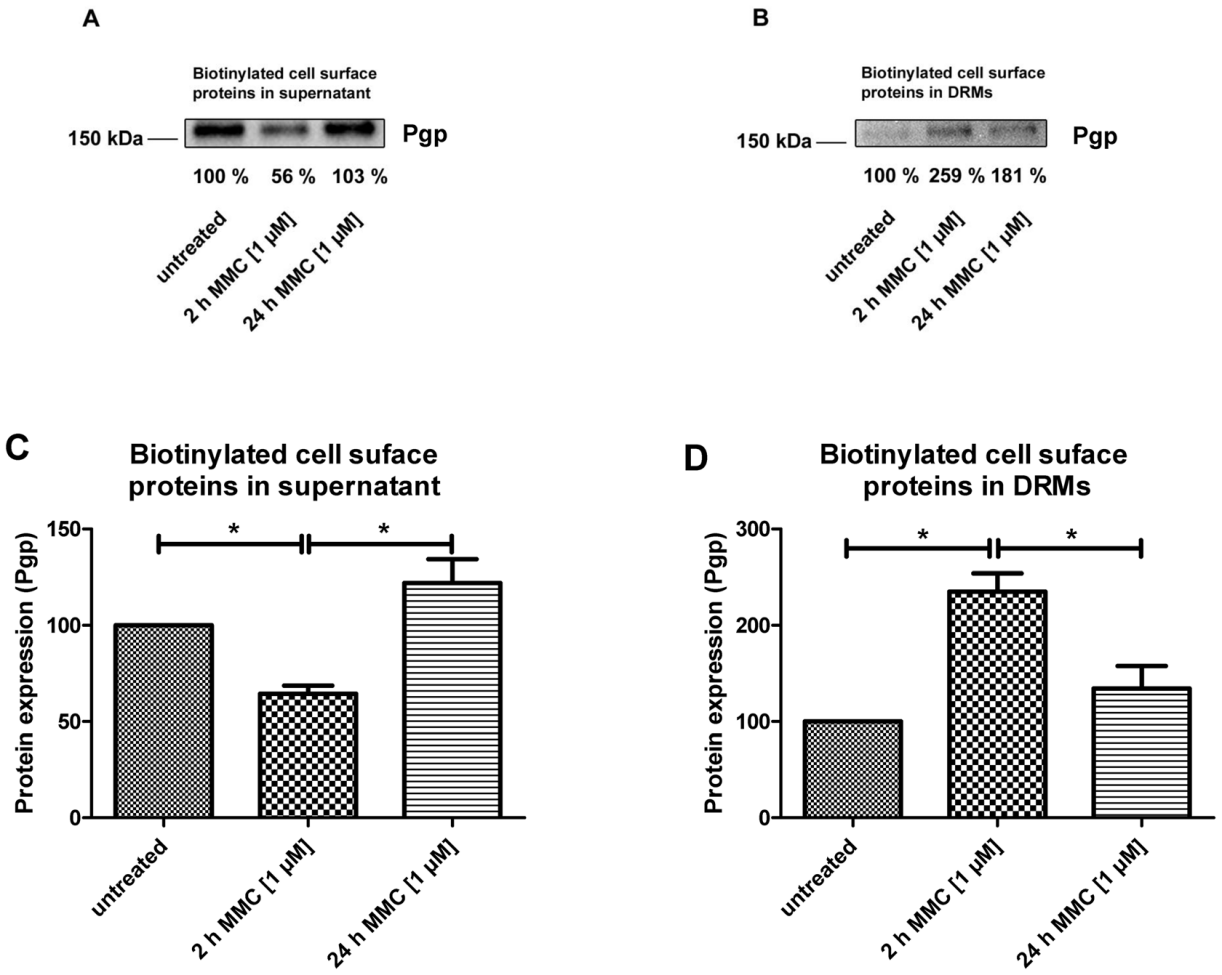
Increased Pgp function (as observed in the uptake assays; see Figures 2, 6, and 7) is associated with the transition of Pgp from detergent (Lubrol) resistant membrane (DRM) domains to detergent soluble membrane domains. Doxycycline-induced hCMEC/D3-MDR1-EGFP cells were treated with MMC (1 µM) for 2 or 4 h and analyzed at the end of the 2 h-exposure period or 20 h after the 4 h-exposure period. Cell surface proteins were biotinylated with EZ-Link Sulfo-NHS-SS-Biotin. After solubilisation of these cells with 1% (w/v) of Lubrol WX, the lysates were centrifuged at 100,000× g for 45 min at 4°C. The DRMs (pellets) resuspended in 0.5 (w/v) DOC and 0.5% (w/v) Triton X-100 and the soluble fractions (supernatant) were subjected to Neutravidin beads to isolate cell surface proteins. These were then analyzed by Western blotting using antibodies against Pgp. The protein bands were analyzed by scanning densitometry. In “A” and “B”, Pgp bands in DRMs and supernatant are presented as percentage of the control in one representative experiment. Data in “C” and “D” are shown as means ± SEM of three experiments. Asterisks denote values that significantly differed (P<0.05).

This temporal disconnect between the Pgp trafficking seen microscopically (Figure 4) and biochemically (Figure 5) and the time to reach measurable alterations in Pgp function prompted us to further address the association of Pgp-EGFP with the cell membrane upon treatment of hCMEC/D3-MDR1-EGFP cells with MMC. As shown before (Figure 4 and Movie S1), 2 h of MMC treatment leads to the assembly of Pgp in punctuate structures at the cell surface. We asked therefore whether these structures are compatible with an association of Pgp with DRMs. For this purpose the cells were biotinylated followed by Lubrol DRM isolation and Western blotting. Figure 8 shows Pgp in the DRM as well as in the Lubrol-soluble fractions. After 2 h of MMC treatment the proportion of Pgp-EGFP protein in the DRM fraction increased by more than 2.5-fold as compared to the control sample (Figure 8B;D). At the same time its proportion in non-DRM or Lubrol-soluble fraction decreased by a similar ratio (Figure 8A;C). Interestingly, the level of Pgp in the DRM fraction decreased after 24 h of MMC treatment with concomitant increase in its proportion in the Lubrol-soluble fraction (Figure 8). The shift of Pgp from the DRM fraction to the detergent-soluble fraction is associated with an increase in the functionality of Pgp as assessed by Rho-123 efflux (Figure 6B and Figure 8).

For direct proof that the temporal disconnect between the trafficking and the time to reach maximum alterations in Pgp function was due to the association of Pgp with DRMs, we disrupted lipid rafts by specifically depleting cholesterol of hCMEC/D3-MDR1-EGFP cells with MβCD. Treatments of these cells with MMC for 2 h led to a significant increase of functional activity of Pgp (Figure 6B). This was correlated with an increase of Pgp in the detergent soluble fraction and a decrease of Pgp in the DRM fraction (see inset in Figure 6B).

## Discussion

In order to study whether Pgp trafficking can be induced in human brain capillary endothelial cells that form the BBB, we transfected hCMEC/D3 cells with a tetracycline-inducible MDR1-EGFP fusion plasmid. MDR1-GFP constructs have been previously used to study intracellular localization and trafficking of Pgp in other cell types [7], [9], [25]–[29], but, to our knowledge, a tetracycline regulatory system to inducibly control Pgp expression has not been previously described. As shown here, Pgp expression can be very effectively controlled in this system, providing an ideal environment in which cellular trafficking of the protein and interactions with a variety of intracellular targets can be studied. We can not rule out that the massive overexpression of the fusion protein changes the physiology of the cells or the localization and trafficking of Pgp, but cell phenotype and viability were not affected, making these transfected cells a useful tool for the aims of the present study.

As known from previous reports [13], [30], [18], hCMEC/D3 cells express endogenous Pgp (and other efflux transporters) but expression of Pgp is lower in hCMEC/D3 cells than in human brain microvessels, although Pgp remains functional as shown by the Rho123 uptake assay [16], [18]. Compared to wild type cells, Rho123 accumulation was significantly decreased in the doxycycline-induced hCMEC/D3-MDR1-EGFP cells, an effect that could be blocked by tariquidar. This observation indicates that the cells were expressing the Pgp-EGFP protein in a functional form on the cell surface, but the extent of this increase in Pgp functionality was smaller than would have been expected from the large (∼15fold) increase in Pgp expression in the transfected cells. This, however, was a consequence of the fact that most of the EGFP-tagged Pgp was localized intracellularly in hCMEC/D3 cells, rendering the transfected cells a valuable tool to study trafficking of Pgp from intracellular pools to the cell membrane.

Newly synthesized Pgp can be delivered to the plasma membrane in different ways [6]. The constitutive pathway involves membrane protein incorporation into transport vesicles which move directly to the plasma membrane along the cytoskeleton [26]. The second pathway is via an intracellular endosomal system in which protein-containing vesicles are transported to endosomal compartments to form an intracellular pool, followed by further transport to the plasma membrane [6]. For Pgp trafficking observed in the present study, the second pathway is relevant, although we do not know yet which endosomal compartment is involved.

For inducing Pgp trafficking in hCMEC/D3 cells, we used MMC, which has previously been reported to induce Pgp trafficking in rat hepatoma (H4IIE) and MDR1-GFP transfected canine kidney (MDCK) cells [7]. The latter study showed that 4 h exposure to subtoxic concentrations of MMC induces an increase in plasma membrane expression and function of Pgp at about 6–12 h after exposure without changing total cellular Pgp. In contrast to various other chemotherapeutic agents, MMC is not a substrate, or at best a very poor substrate, for Pgp and does not induce MDR1 mRNA [31], so that Pgp trafficking might be the only way by which the cell can protect itself against this toxic compound. However, the mechanisms by which MMC induces Pgp trafficking are not known. In the present study, MMC was shown to induce Pgp trafficking in MDR1-EGFP transfected hCMEC/D3 cells, which, to our knowledge, is the first direct evidence that drug-induced Pgp trafficking occurs in brain capillary endothelial cells. Pgp trafficking was unequivocally demonstrated by using confocal laser scanning microscopy after exposure to MMC. Furthermore, similar to the findings of Maitra et al. [7] in other cell types, MMC significantly increased membrane-associated Pgp (but not total cellular Pgp) and increased Pgp functionality as demonstrated by decreased Rho123, eFluxx-ID Gold, and calcein accumulation. The most marked effect of MMC exposure was obtained with the relatively new eFluxx-ID Gold assay, in which only viable cells are analyzed by flow cytometry [19], [20]. These data demonstrate for the first time that endothelial cells that form the human BBB respond with Pgp trafficking to exposure with a potentially toxic compound.

Cellular uptake of the fluorescent Pgp substrates Rho123 and calcein-AM has previously been used to measure the functionality of Pgp in hCMEC/D3 cells [13], [16]. By using these assays with and without inhibitors of Pgp, multidrug resistance associated proteins (MRPs) and breast cancer resistance protein (BCRP), Rho123 was more sensitive and selective to demonstrate Pgp functionality in these cells than calcein-AM [13], which is supported by the present experiments with tariquidar and MMC in transfected hCMEC/D3 cells.

While Pgp trafficking occurred relatively rapidly after exposure to MMC, it took more than 12 h before a significant decrease in Rho123 or eFluxx-ID Gold uptake was determined, indicating a temporal disconnect between the trafficking and the time to reach a significant increase in Pgp function. This temporal discrepancy between the confocal microscopic and Western blot findings and Pgp substrate uptake measurements in the present MMC experiments suggests that the bulk of Pgp is transported and clustered in membrane domains and this type of Pgp assembly is associated with a decrease in Pgp function, whereby the release of Pgp from these domains occurs only slowly. This hypothesis is supported by the localization pattern and association of Pgp with DRMs or lipid rafts [11] and the increased functionality of Pgp following disruption of lipid rafts by MβCD. The fact that no comparable trafficking of Pgp-EGFP to the membrane was observed with confocal microscopy under control conditions (i.e., in the absence of MMC) can be explained by previous studies in MDR1-EGFP transfected HeLa cells, which demonstrated that 12–48 h are required before Pgp-EGFP is transported to the plasma membrane [9], i.e., much longer than observed in this study with MMC.

Drug-induced trafficking of Pgp at the BBB has previously been suggested by Ott et al. [32] by studying effects of St. John's Wort (SJW) and SJW constituents on Pgp transport activity in porcine brain capillary endothelial cells and freshly isolated brain capillaries from pigs. The SJW constituent quercetin enhanced Pgp transport activity (as indicated by calcein-AM uptake), which could be blocked by brefeldin A, an inhibitor of vesicular trafficking between the endoplasmatic reticulum and the Golgi apparatus [32]. Based on this observation, Ott et al. [32] proposed that the increase in Pgp activity by quercetin was likely due to trafficking and membrane insertion of sub-apical vesicles containing transporter protein, but no direct evidence for this hypothesis was provided. Likewise, Hartz et al. [33] suggested that protein kinase C (PKC) may influence Pgp trafficking in brain capillary endothelial cells, stimulating retrieval from the plasma membrane into a vesicular compartment, as has been demonstrated for Pgp in hepatocytes [34], but, again, no direct evidence has been provided. A recent study by McCaffrey et al. [11] demonstrated induction of Pgp trafficking at the BBB by peripheral inflammatory pain in rats, and indicated that this stimulus promotes a dynamic redistribution between membrane domains of Pgp and caveolin-1.

At the BBB, Pgp is mainly localized in caveolin-1/flotillin-2 positive caveolar microdomains (known as lipid rafts) and its activity can be modulated by interaction with caveolin-1 [35]. This localization in microdomains most likely explains the clustered formation of Pgp-EGFP observed in our study and a more recent study by Huber et al. [20] in hCMEC/D3 cells. The mechanism by which the Pgp activity is induced by MMC is not entirely clear. However, our data reveal an MMC-induced time-dependent punctuate pattern of Pgp at the cell surface. This pattern is compatible with the association of Pgp with Lubrol DRMs. Interestingly, reduced levels of Pgp in Lubrol DRMs and its concomitant increase in the Lubrol-soluble fraction are associated with increased function, and such increased function is also observed at earlier time points when disrupting lipid rafts by MβCD.

Certainly, it would be tempting to study the effects of other compounds, including antiepileptic drugs [36] and neurotoxic compounds such as glutamate, on Pgp trafficking in MDR1-EGFP-transfected hCMEC/D3 cells. In addition to better understanding how Pgp at the BBB responds to drug exposure, manipulation of Pgp trafficking, e.g., by preventing its release from subcellular stores, may present a novel, reversible means of modulating drug delivery to the brain.

## Supporting Information

Figure S1 **Concentration-dependent increase in Pgp functionality by doxycycline in hCMEC/D3-MDR1-EGFP cells.** The Rho123 uptake assay was used for determining Pgp functionality. Data are shown as means ± SEM of three experiments per doxycycline concentration. Asterisks denote values that significantly differ from Rho123 uptake in the absence of doxycycline (P<0.01).
(TIF)

Figure S2 **Efflux of rhodamine 123 (Rho123) in doxycycline-induced hCMEC/D3-MDR1-EGFP cells in the absence (“untreated”) or presence of the Pgp inhibitor tariquidar (TQ; 0.5 µM).** “A”: Cells were preloaded with Rho123 at 4°C (to inhibit any Pgp-mediated efflux) and then the decay of intracellular fluorescence was measured at 37°C at 15, 30 and 60 min following preloading. Data are shown as means ± SEM (n = 3). The Rho123 efflux rate as calculated from these data is shown in “B”. Significant differences between untreated and tariquidar-treated cells is indicated by asterisk (P<0.05).
(TIF)

Figure S3 **Determination of transport rates and effect of MMC in the calcein AM extrusion assay in the absence or presence of tariquidar.** Efflux of calcein AM was indirectly measured by determining the intracellular concentration of the fluorescent calcein at different concentrations (0.25, 0.5, 1, and 2 µM) of calcein AM. The transport rate was calculated in calcein fluorescence per sec per mg protein. In the MMC experiments, transport was measured 20 h after a 4 h exposure with 1 µM MMC. Although the effect size was small, MMC significantly decreased intracellular calcein at all concentrations of calcein AM (indicated by rhomb; P<0.05), demonstrating enhanced calcein AM efflux. Tariquidar (TQ; 0.5 µM) completely counteracted this effect of MMC (indicated by asterisk; P<0.001). Data are shown as means ± SEM (n = 3).
(TIF)

Movie S1 **Mitomycin C induced Pgp-EGFP trafficking in hCMEC/D3-MDR1-EGFP (doxycycline-on) cells.** The movie shows a period of 79.2 min in fast motion following 1 h of MMC exposure (see Figure 4 for single images and more details). In the endothelial cell in the centre of the images, we observed that Pgp-EGFP trafficked from intracellular compartments to the membrane; this started within 15 min after onset of confocal microscopic analysis (i.e., about 75 min after onset of MMC exposure).
(ZIP)

Movie S2 **Lack of obvious Pgp-EGFP trafficking in hCMEC/D3-MDR1-EGFP (doxycycline-on) cells in the absence of mitomycin C.** The same time frame as used in Movie S1 is used.
(ZIP)
